# Drug survival of IL‐23 and IL‐17 inhibitors versus other biologics for psoriasis: A British Association of Dermatologists Biologics and Immunomodulators Register cohort study

**DOI:** 10.1111/jdv.20739

**Published:** 2025-05-29

**Authors:** Leila Motedayen Aval, Zenas Z. N. Yiu, Oras A. Alabas, Christopher E. M. Griffiths, Nick J. Reynolds, Philip J. Hampton, Catherine H. Smith, Richard B. Warren, BADBIR Study Group

**Affiliations:** ^1^ Division of Musculoskeletal and Dermatological Sciences, School of Biological Sciences, Faculty of Biology, Medicine and Health, The University of Manchester The Dermatology Centre, Salford Royal Hospital, Northern Care Alliance NHS Foundation Trust, Manchester Academic Health Science Centre Manchester UK; ^2^ Department of Dermatology, King's College Hospital King's College London London UK; ^3^ Department of Dermatology, Royal Victoria Infirmary and NIHR Newcastle Biomedical Research Centre, Newcastle Hospitals NHS Foundation Trust Institute of Translational and Clinical Medicine, Medical School, Newcastle University Newcastle upon Tyne UK; ^4^ St John's Institute of Dermatology, Guy's and St Thomas' NHS Foundation Trust, and King's College London London UK

## Abstract

**Background:**

Interleukin (IL)‐23p19 and IL‐17 inhibitors have demonstrated high efficacy for psoriasis in randomized controlled trials, though real‐world data, particularly for risankizumab (IL‐23p19 inhibitor) and brodalumab (IL‐17 receptor (IL‐17R) inhibitor), is limited.

**Objectives:**

To assess drug survival of IL‐23p19 and IL‐17 inhibitors compared to other biologics for psoriasis.

**Methods:**

We conducted a cohort study using data from the British Association of Dermatologists Biologics and Immunomodulators Register (BADBIR) from November 2007 to June 2023. Multivariable flexible parametric models assessed drug survival, with discontinuation due to ineffectiveness and adverse effects reported separately. The primary outcome measure was the absolute difference in restricted mean survival time at 2 years, referred to as adjusted survival time, between all comparators.

**Results:**

Among 19,034 treatment courses (median follow‐up: 2.3 years), treatments included adalimumab (tumour necrosis factor‐alpha (TNF‐a) inhibitor, *n* = 6,815), ustekinumab (IL‐12/23p40 inhibitor, *n* = 5,639), secukinumab (IL‐17A inhibitor, *n* = 3,051), ixekizumab (IL‐17A inhibitor, *n* = 1,072), brodalumab (*n* = 367), guselkumab (IL‐23p19 inhibitor, *n* = 1,258) and risankizumab (*n* = 832).

Guselkumab and risankizumab had the highest adjusted survival times (years [interquartile ranges]) for effectiveness (1.93 [1.91–1.95] and 1.93 [1.90–1.96], respectively). Risankizumab had the highest survival for safety (1.94 [1.92–1.96]) followed by guselkumab (1.92 [1.90–1.94]) and ustekinumab (1.92 [1.91–1.93]). Brodalumab showed lower adjusted survival time for effectiveness (1.75 [1.69–1.81]) than most biologics except secukinumab and adalimumab; and similar survival for safety (1.85 [1.81–1.90]) compared to IL‐17A inhibitors and adalimumab. In patients with psoriatic arthritis, ustekinumab showed reduced drug survival. Prior biologic exposure was associated with a dose–response reduction in survival which was significantly larger for IL‐17 inhibitors.

**Conclusions:**

Guselkumab and risankizumab have the most favourable drug survival for effectiveness, with comparable safety to ustekinumab, and more favourable than other BADBIR biologics. Longer drug survival may reduce treatment burden by minimizing treatment switches, clinic visits and disease flares, supporting IL‐23p19 inhibitors as a practical long‐term option for psoriasis.

## Graphical abstract

Psoriasis affects 2–3% of the population, with numerous biologic options complicating treatment selection. This real‐world study found IL‐23p19 inhibitors offer the longest‐lasting effectiveness and safety. Psoriatic arthritis reduced ustekinumab survival, and prior biologic use impacted persistence, especially for IL‐17 inhibitors. These findings support more personalized, long‐term psoriasis treatment choices.

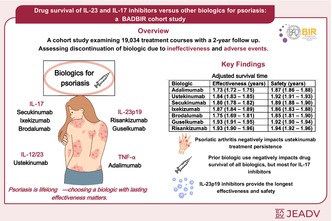

**Why was the study undertaken?**

This study was undertaken to address gaps left by randomized controlled trials in comparing the effectiveness of IL‐23p19 and IL‐17 inhibitors, specifically risankizumab and brodalumab, against each other in a representative clinical population.

**What does this study add?**

We showed that IL‐23p19 inhibitors have the highest drug survival, with patients persisting 21weeks longer for effectiveness and 13weeks for safety over 2 years. For the first time, we report brodalumab's drug survival, demonstrating comparable effectiveness and safety to adalimumab and secukinumab.

**What are the implications of this study for disease understanding and/or clinical care?**

This study informs biologic choice by highlighting IL‐23p19 inhibitors' extended drug survival, psoriatic arthritis' negative effect on ustekinumab, and the impact of prior treatment on drug persistence, helping clinicians tailor treatments for patients prioritizing long‐term efficacy and safety.

## INTRODUCTION

Network meta‐analyses of randomized controlled trials (RCTs) have shown that newer therapies targeting interleukin (IL)‐23 p19 and IL‐17 are more efficacious than the older biologics such as tumour necrosis factor (TNF)‐α inhibitors and ustekinumab, an IL‐12/23 inhibitor, for the treatment of psoriasis.^1^ RCTs are fundamental to guiding clinical practice as the gold standard for investigating treatment efficacy but can be restricted by sample size, follow‐up duration, and stringent inclusion–exclusion criteria, leading to an efficacy‐effectiveness gap with poorer treatment outcomes seen in clinic.^2^, ^3^ For example, 29.8% of patients from a Spanish dermatology biologics registry would be ineligible for psoriasis biologic clinical trials based on common trial eligibility criteria; pertinently, the ineligible patients were found to be at increased risk of serious adverse events.^2^ Real‐world evidence complements RCTs and helps to bridge the efficacy‐effectiveness gap by assessing treatment effect in more heterogeneous populations under routine clinical settings.

Drug survival refers to the duration of time between initiating and stopping medication and is a proxy measure for the effectiveness, safety, adherence, and tolerability of a treatment.^4^ We previously reported on drug survival of adalimumab, guselkumab, ixekizumab, secukinumab and ustekinumab in patients with psoriasis using data from the British Association of Dermatologists Biologics and Immunomodulators (BADBIR), a large national pharmacovigilance register of UK and Republic of Ireland (RoI) patients with psoriasis on systemic therapy, and identified that guselkumab had the highest drug survival for effectiveness out of the comparators.^5^

To date, few studies have reported on the drug survival of risankizumab and brodalumab.^6^, ^7^, ^8^, ^9^, ^10^, ^11^, ^12^ Limitations of these studies include relatively small sample sizes or single‐arm studies for brodalumab and risankizumab; difficulties in interpreting overall drug survival without reasons for discontinuation and methodological problems, such as not accounting for non‐proportional hazards for modelling and interpretation.^13^

Our aim for this cohort study was to compare the drug survival of all current commonly used biologics for moderate to severe psoriasis in BADBIR, including brodalumab and risankizumab.

## MATERIALS AND METHODS

### Data source and study population

The structure, study design, and baseline characteristics of BADBIR have been documented previously.^14^, ^15^ BADBIR, a large observational pharmacovigilance study established in September 2007, assesses the long‐term safety of biologics in psoriasis patients in the UK and Eire. Over 20,000 patients from 167 secondary care dermatology centres are registered and are followed up bi‐annually for 3 years and annually thereafter. Baseline and follow‐up visits record demographic and anthropometric data, comorbidities, and treatment information. BADBIR was approved by the National Health Service Research Ethics Committee North‐West England (07/MRE08/9), with all participants providing written informed consent per the Declaration of Helsinki.

Data from November 2007 to June 2023 were used. Eligible patients were adults with chronic plaque psoriasis and were recruited or switched into the biologic cohort after initiating treatment with either TNF‐α inhibitor adalimumab (Humira; AbbVie), IL‐12/23p40 inhibitor ustekinumab (Stelara; Janssen), IL‐17A inhibitors secukinumab (Cosentyx; Novartis) and ixekizumab (Taltz; Lilly), IL‐17 receptor (IL‐17R) inhibitor brodalumab (Kyntheum; LEO Pharma) or IL‐23p19 inhibitors guselkumab (Tremfya; Janssen) and risankizumab (Skyrizi; AbbVie). We included patients who received any of these therapies with at least one available follow‐up record, counting each treatment course separately.

### Data analysis

We defined discontinuation of therapy as a gap between stop date and restart date exceeding 90 days, with recorded reason for discontinuation entered by the clinical team as due to ineffectiveness; the occurrence of adverse events (AEs) or other reasons. We censored patients at the last available follow‐up date. We conducted a descriptive summary of the baseline characteristics for the biologic cohorts. Unadjusted Kaplan–Meier survival analysis and survival functions at 1 and 2 years were calculated for each biologic and for overall discontinuation; discontinuation due to ineffectiveness and discontinuation due to AEs separately.

### Model development

We developed two models for biologic discontinuation: one for ineffectiveness (effectiveness model) and one for AEs (safety model). An interaction with time was fitted in both models to account for non‐proportional hazards. We used a two‐tier predictor selection process.^5^ In the first tier, we included covariates consistently associated with biologic drug survival, which were age, sex, body mass index, previous biologic treatments (a categorical variable of 0 for first‐line therapy, 1 for second‐line therapy and 2 for third or further lines of therapy) and psoriatic arthritis (PsA) for the effectiveness model; and age and sex for the safety model. Second‐tier covariates were chosen based on a priori considerations from our previous studies and a systematic review.^16^ These included ethnicity (Asian [Chinese, Indian and Pakistani], White and Other minority groups [which included Black British individuals]), baseline psoriasis area and severity index (PASI), smoking, alcohol intake, chronic obstructive pulmonary disease, type 1 diabetes, dyslipidemia, waist circumference, number of comorbid conditions, time‐dependent concomitant use of methotrexate or cyclosporine, nail psoriasis, palmoplantar psoriasis, flexural psoriasis, scalp psoriasis and unstable psoriasis. Biologics were included as a categorical variable.

Using the same methodology as our previous work,^5^ we applied flexible parametric models (FPMs) using the stpm3 command in Stata, allowing for non‐proportional effects of covariates.^17^ The selection of knots for the restricted cubic spline function was based on minimizing the Akaike information criterion and Bayesian information criterion. We employed 20 multiply imputed datasets for missing data. We used the mfpmi command in Stata to test second‐tier covariates for inclusion using backward stepwise regression (p‐value of 0.10 as the cutoff) and to transform continuous predictors using fractional polynomials to address nonlinearity.^18^ We investigated potential effect modification using likelihood ratio tests for selected categorical covariates.

We reported the output from the effectiveness and safety models in three different ways. Given that the Kaplan–Meier survival curves for the biologics crossed over time, indicating non‐proportional hazards, we employed FPMs. The commonly used Cox proportional hazards model assumes a constant hazard ratio over time, which would be incorrect in this setting. FPMs explicitly model the baseline hazard and allow for time‐dependent interactions. We reported hazard ratios using ustekinumab as an arbitrary anchor comparator. However, the hazard ratios cannot be interpreted alone as time‐varying relative difference in hazard is considered by the interaction term between biologic and time. The shape of the relative hazard over time, which may change given the likely non‐proportional hazards between the comparators, is not reflected in a single hazard ratio number.^19^

To address this, we also calculated restricted mean survival time (RMST) at 2 years for each biologic and the difference in RMST between all comparator biologics. RMST is a quantitative measure of the absolute difference in survival time between groups, representing the average duration of drug survival over a restricted follow‐up period.^19^ Unlike hazard ratios, RMST does not rely on the proportional hazards assumption, making it a more robust and clinically interpretable metric when hazards vary over time. We chose 2 years as the follow‐up period, as this is the closest whole digit to the median follow‐up time. We refer to RMST as adjusted survival time. For data visualization, we plotted population‐averaged standardized survival curves, which were overlaid with the corresponding crude Kaplan–Meier curves.

To understand our previous finding of a drop in drug survival for ixekizumab and secukinumab in people with prior biologics exposure, we conducted sensitivity analyses to evaluate whether discontinuation due to ineffectiveness of either previous TNF inhibitors (TNFi), ustekinumab or IL‐17 inhibitors was associated with any difference in drug survival in people starting ixekizumab, secukinumab or brodalumab as third or subsequent line of therapy; previous exposure to IL‐23p19 inhibitors was not evaluated as the sample size was too low. All analyses were performed using Stata, version 17.0 (StataCorp). The study was reported according to Strengthening the Reporting of Observational Studies in Epidemiology (STROBE) guidelines.

## RESULTS

### Baseline characteristics and cohort overview

A total of 19,034 treatment courses were eligible for inclusion, with 6,815 (35.8%) receiving adalimumab, 5,639 (29.6%) ustekinumab, 3,051(16.0%) secukinumab, 1,258(6.6%) guselkumab, 1,072 (5.6%) ixekizumab, 367 (1.9%) brodalumab and 832 (4.4%) risankizumab. The overall median age at therapy initiation was 48.0 years (interquartile range (IQR), 46.0–49.0), with a median BMI of 31.0 (IQR, 30.7–31.9), and a median PASI of 11.4 (IQR, 11.1–12.5). The baseline characteristics of the cohort separated by biologic therapy are presented in Table 1. Demographic data correspond to each initiation timepoint so one patient may appear multiple times.

**TABLE 1:** Baseline demographic and disease characteristics of the biologic cohorts.

	Secukinumab	Adalimumab	Brodalumab	Risankizumab	Ustekinumab	Ixekizumab	Guselkumab
	*N* = 3051	*N* = 6815	*N* = 367	*N* = 832	*N* = 5639	*N* = 1072	*N* = 1258
Age, median (IQR), y	47.0 (37.0–56.0)	45.0 (35.0–54.0)	49.0 (39.0–57.0)	49.0 (38.0–57.0)	46.0 (36.0–55.0)	48.0 (37.0–57.0)	48.5 (38.0–57.0)
Female sex	1333 (43.7%)	2850 (41.8%)	152 (41.4%)	371 (44.6%)	2398 (42.5%)	478 (44.6%)	542 (43.1%)
Ethnicity							
White	2254 (73.9%)	5417 (79.5%)	253 (68.9%)	543 (65.3%)	4521 (80.2%)	747 (69.7%)	843 (67.0%)
Asian	180 (5.9%)	274 (4.0%)	19 (5.2%)	53 (6.4%)	283 (5.0%)	59 (5.5%)	73 (5.8%)
Other^a^	127 (4.2%)	237 (3.5%)	13 (3.5%)	35 (4.2%)	208 (3.7%)	51 (4.8%)	58 (4.6%)
Missing^b^	490 (16.1%)	887 (13.0%)	82 (22.3%)	201 (24.2%)	627 (11.1%)	215 (20.1%)	284 (22.6%)
BMI (kg/m^2^), Median (IQR)	31.0 (27.2–36.1)	29.8 (26.1–34.4)	31.9 (27.9–37.4)	30.7 (27.0–36.1)	30.8 (26.4–36.0)	31.9 (27.4–37.1)	31.4 (27.5–37.0)
Missing	838 (27.5%)	1285 (18.9%)	159 (43.3%)	418 (50.2%)	1110 (19.7%)	391 (36.5%)	557 (44.3%)
Waist circumference (cm), Median (IQR)	103.0 (93.0–115.0)	100.0 (90.0–111.0)	106.0 (97.0–120.0)	102.0 (92.0–113.0)	103.0 (91.0–114.0)	104.0 (94.0–115.0)	104.0 (94.0–117.0)
Missing	1135 (37.2%)	1578 (23.2%)	206 (56.1%)	542 (65.1%)	1505 (26.7%)	514 (48.0%)	669 (53.2%)
Alcohol intake by categories							
No documented alcohol intake	781 (25.6%)	1799 (26.4%)	75 (20.4%)	148 (17.8%)	1641 (29.1%)	224 (20.9%)	238 (18.9%)
Lower risk drink (<21 U/WK for men, <14 U/WK for women)	918 (30.1%)	2741 (40.2%)	71 (19.3%)	159 (19.1%)	2104 (37.3%)	243 (22.7%)	261 (20.7%)
Hazardous drinking (<50 U/WK for men, <35 U/WK for women)	196 (6.4%)	597 (8.8%)	27 (7.4%)	46 (5.5%)	418 (7.4%)	44 (4.1%)	66 (5.2%)
Harmful drinking (≥50 U/WK for men, ≥35 U/WK for women)	22 (0.7%)	89 (1.3%)	2 (0.5%)	8 (1.0%)	70 (1.2%)	9 (0.8%)	7 (0.6%)
Missing^c^	1134 (37.2%)	1589 (23.3%)	192 (52.3%)	471 (56.6%)	1406 (24.9%)	552 (51.5%)	686 (54.5%)
Smoking status							
Never smoked	1444 (47.3%)	3190 (46.8%)	178 (48.5%)	447 (53.7%)	2437 (43.2%)	512 (47.8%)	620 (49.3%)
Previous smoker	964 (31.6%)	2092 (30.7%)	114 (31.1%)	254 (30.5%)	1838 (32.6%)	330 (30.8%)	410 (32.6%)
Current smoker	643 (21.1%)	1533 (22.5%)	75 (20.4%)	131 (15.7%)	1364 (24.2%)	230 (21.5%)	228 (18.1%)
Disease duration, median, (IQR), y	20.0 (12.0–31.0)	20.0 (12.0–30.0)	21.0 (12.0–31.0)	22.0 (14.0–33.0)	20.0 (12.0–30.0)	21.0 (14.0–31.0)	21.0 (13.0–32.0)
Baseline DLQI	16.0 (11.0–22.0)	17.0 (11.0–23.0)	14.0 (7.0–22.0)	15.5 (9.0–22.0)	16.0 (10.0–22.0)	16.0 (9.0–24.0)	15.0 (9.0–21.0)
Missing	1661 (54.4%)	3189 (46.8%)	222 (60.5%)	566 (68.0%)	2806 (49.8%)	685 (63.9%)	797 (63.4%)
Baseline PASI	12.0 (7.8–17.4)	13.2 (10.2–18.4)	11.1 (7.2–15.3)	11.0 (7.0–16.0)	12.5 (8.9–17.7)	11.4 (6.9–17.1)	11.1 (6.9–16.2)
Missing	620 (20.3%)	996 (14.6%)	65 (17.7%)	200 (24.0%)	887 (15.7%)	236 (22.0%)	284 (22.6%)
Psoriatic arthritis (any)	1066 (34.9%)	1924 (28.2%)	98 (26.7%)	155 (18.6%)	1279 (22.7%)	446 (41.6%)	331 (26.3%)
Psoriasis							
Nails	1606 (52.6%)	3794 (55.7%)	187 (51.0%)	421 (50.6%)	2966 (52.6%)	601 (56.1%)	627 (49.8%)
Palmoplantar	590 (19.3%)	1273 (18.7%)	71 (19.3%)	145 (17.4%)	1098 (19.5%)	225 (21.0%)	244 (19.4%)
Scalp	2132 (69.9%)	4811 (70.6%)	257 (70.0%)	587 (70.6%)	4034 (71.5%)	766 (71.5%)	875 (69.6%)
Unstable	359 (11.8%)	779 (11.4%)	33 (9.0%)	62 (7.5%)	613 (10.9%)	128 (11.9%)	149 (11.8%)
Flexural	1065 (34.9%)	2538 (37.2%)	145 (39.5%)	284 (34.1%)	2114 (37.5%)	419 (39.1%)	469 (37.3%)
No. of previous biologic therapies							
None	1043 (34.2%)	4978 (73.0%)	73 (19.9%)	199 (23.9%)	2519 (44.7%)	205 (19.1%)	290 (23.1%)
1	1059 (34.7%)	1417 (20.8%)	93 (25.3%)	298 (35.8%)	2153 (38.2%)	334 (31.2%)	379 (30.1%)
≥2	949 (31.1%)	420 (6.2%)	201 (54.8%)	335 (40.3%)	967 (17.1%)	533 (49.7%)	589 (46.8%)
During follow up							
Any treatment with methotrexate	342 (11.2%)	1035 (15.2%)	26 (7.1%)	37 (4.4%)	601 (10.7%)	92 (8.6%)	70 (5.6%)
Any treatment with cyclosporine	89 (2.9%)	350 (5.1%)	12 (3.3%)	17 (2.0%)	247 (4.4%)	36 (3.4%)	24 (1.9%)
No. of comorbid conditions							
None	732 (24.0%)	1650 (24.2%)	94 (25.6%)	181 (21.8%)	1477 (26.2%)	206 (19.2%)	336 (26.7%)
1–2	1658 (54.3%)	3753 (55.1%)	183 (49.9%)	440 (52.9%)	2847 (50.5%)	572 (53.4%)	620 (49.3%)
3–4	535 (17.5%)	1172 (17.2%)	74 (20.2%)	171 (20.6%)	1048 (18.6%)	247 (23.0%)	244 (19.4%)
≥5	126 (4.1%)	240 (3.5%)	16 (4.4%)	40 (4.8%)	267 (4.7%)	47 (4.4%)	58 (4.6%)
COPD	64 (2.1%)	136 (2.0%)	9 (2.5%)	10 (1.2%)	150 (2.7%)	21 (2.0%)	30 (2.4%)
Hypertension	729 (23.9%)	1588 (23.3%)	102 (27.8%)	226 (27.2%)	1351 (24.0%)	262 (24.4%)	293 (23.3%)
Diabetes	332 (10.9%)	584 (8.6%)	47 (12.8%)	102 (12.3%)	595 (10.6%)	128 (11.9%)	151 (12.0%)
Dyslipidaemia	244 (8.0%)	653 (9.6%)	35 (9.5%)	69 (8.3%)	546 (9.7%)	114 (10.6%)	102 (8.1%)

Abbreviations: BMI, body mass index (calculated as weight in kilograms divided by height in metres squared); COPD, chronic obstructive pulmonary disease; DLQI, Dermatology Life Quality Index; PASI, baseline psoriasis area and severity index; IQR, interquartile range.

^a^ Other included Black British Individuals.

^b^ The amount of missing data given for covariates with missing data.

The overall median follow‐up time was 2.3 years. Ustekinumab, with a median of 3.3 years (IQR, 1.4–5.8), had the longest accrued follow‐up time, followed by adalimumab (median, 2.4 [IQR, 0.9, 4.9 years]), secukinumab (median, 2.2 [IQR, 1.0, 4.0 years]), brodalumab (median, 1.7 [IQR, 0.6, 3.0 years]), guselkumab (median, 1.7 [IQR, 0.8, 2.9 years]), ixekizumab (median, 1.5 [IQR, 0.7, 2.9 years]) and risankizumab (median, 1.0 [IQR, 0.5, 1.7 years]). The survival functions for the biologic cohorts are listed in Table S1. Guselkumab and risankizumab demonstrated the highest crude drug survival across outcomes.

### Flexible parametric modeling results

Flexible parametric models were used to estimate drug survival outcomes, with hazard ratios for ineffectiveness and AE‐related discontinuation presented in Tables S2 and S3. PsA and the number of previous biologics were significant effect modifiers in the effectiveness model, indicating that these factors influenced treatment persistence. However, no significant effect modifiers were identified in the safety model. Adjusted drug survival times (RMST at 2 years) for effectiveness and safety across biologics are presented in Table 2.

**TABLE 2:** Restricted mean survival time at 2 years for each of the biologics for the effectiveness and safety models.

Biologic	Effectiveness (years)	Safety (years)
Adalimumab	1.73 (1.72–1.75)	1.87 (1.86–1.88)
Ustekinumab	1.84 (1.83–1.85)	1.92 (1.91–1.93)
Secukinumab	1.80 (1.78–1.82)	1.89 (1.88–1.90)
Ixekizumab	1.87 (1.84–1.89)	1.86 (1.83–1.88)
Brodalumab	1.75 (1.69–1.81)	1.85 (1.81–1.90)
Guselkumab	1.93 (1.91–1.95)	1.92 (1.90–1.94)
Risankizumab	1.93 (1.90–1.96)	1.94 (1.92–1.96)

*Note*: Each cell contains the restricted mean survival time over 2 years with corresponding 95% confidence interval for the outcomes of effectiveness and safety for the intervention.

### Drug survival by effectiveness and safety

Guselkumab and risankizumab demonstrated the highest adjusted survival time for effectiveness (both RMST: 1.93 years, 95% CI: 1.91–1.95 and 1.90–1.96, respectively) (Table 2). For safety, risankizumab showed the highest survival (1.94 years, 95% CI 1.92–1.96, years) followed closely by guselkumab (1.92 years, 95% CI 1.90–1.94, years) and ustekinumab (1.92 years, 95% CI 1.91–1.93, years) (Table 2). Absolute differences in RMST between biologics are detailed in Table 3.

**TABLE 3:** Standardized difference (Cohen's *d*) in restricted mean survival time at 2 years between biologics for effectiveness and safety.

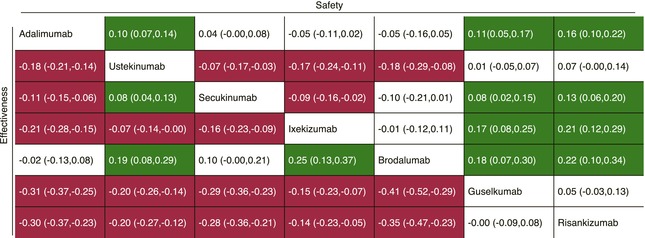

*Note*: Each cell contains the standardized mean difference and 95% confidence interval between the restricted mean survival time over 2 years for the two outcomes of effectiveness and safety of the intervention in the respective column versus the comparator in the respective row. The cells shaded in green show superiority for the intervention; red shows inferiority for the intervention and white shows no statistical difference between the intervention and the comparator.

### Comparison between biologics

**FIGURE 1:**
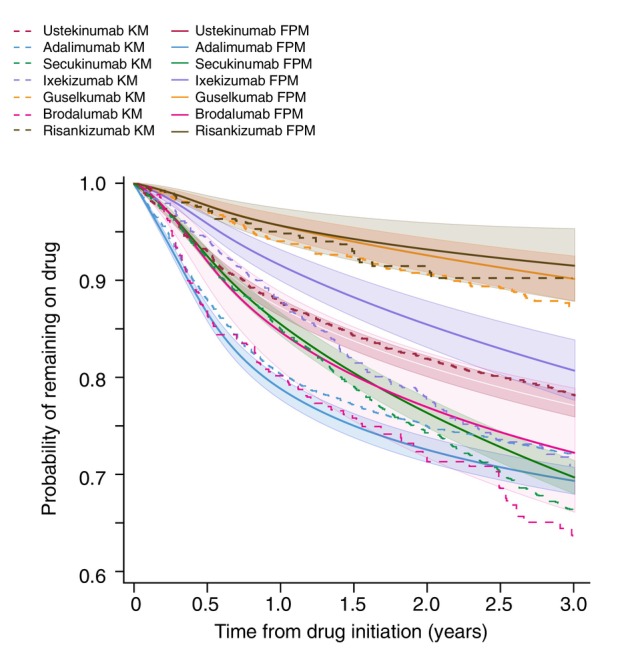
Discontinuation due to ineffectiveness: Overlaid Kaplan–Meier (KM) survival curves and Flexible Parametric Model (FPM) survival curves for discontinuation due to ineffectiveness over 2 years. FPM estimates survival using restricted cubic splines, allowing for time‐dependent hazards and more accurate drug survival comparisons. Shaded areas represent 95% confidence intervals for the FPM estimates. The *y*‐axis starts from 0.60 for presentation clarity purposes.

**FIGURE 2:**
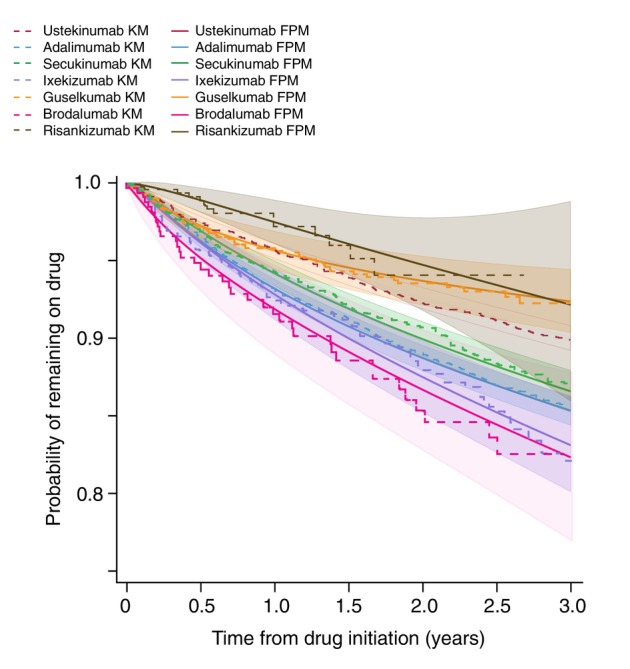
Discontinuation due to adverse events: Overlaid Kaplan–Meier (KM) survival curves and Flexible Parametric Model (FPM) survival curves for discontinuation due to adverse events over 2 years. FPM accounts for changes in treatment persistence over time and provides restricted mean survival time (RMST) estimates for improved clinical interpretation. Shaded areas represent 95% confidence intervals for the FPM estimates. *Y*‐axis starts from 0.75 for presentation clarity purposes.

To further compare biologic therapies, Table 3 presents the absolute differences (Cohen's *d*) in RMST at 2 years for effectiveness and safety. Guselkumab and risankizumab had significantly higher adjusted drug survival for effectiveness compared to all biologics except each other. Similarly, for safety, both demonstrated superior survival times relative to all biologics except ustekinumab (Table 3). Among IL‐17 inhibitors, secukinumab exhibited significantly lower adjusted drug survival than ixekizumab for effectiveness but had a significantly higher safety profile. Brodalumab showed significantly lower adjusted drug survival for effectiveness relative to all biologics except adalimumab and secukinumab and lower drug survival for safety compared to all biologics except the IL‐17A inhibitors and adalimumab. These findings are visualized in the population‐averaged survival curves presented in Figure 1 (effectiveness) and Figure 2 (safety).

### Impact of psoriatic arthritis (PsA)

Stratified analyses demonstrated that PsA significantly impacted drug survival. In patients without PsA, ustekinumab showed higher adjusted drug survival than secukinumab (RMST difference 0.11 years, 95% CI 0.06–0.17 years) and comparable survival to ixekizumab (−0.04 years, 95% CI −0.012 to 0.05). In contrast, in PsA patients, ustekinumab's survival was comparable to secukinumab (−0.06, 95% CI −0.14 to 0.02) but lower than ixekizumab (−0.17, 95% CI −0.28 to −0.06). Adjusted drug survival at 2 years and the population‐averaged survival curves for the model stratified for PsA are presented in Table S4 and Figure S1. All other comparisons did not differ significantly from the overall model in Table 3.

### Impact of prior biologic use

Biologic survival varied across treatment lines, with IL‐17 inhibitors displaying the most notable declines. In first‐line therapy, secukinumab had significantly higher adjusted drug survival compared to adalimumab and similar drug survival to ustekinumab, but this declined in subsequent lines of therapy (Table S5). Similarly, ixekizumab initially outperformed adalimumab and ustekinumab and demonstrated similar survival to guselkumab and risankizumab for first‐line therapy (Figure S2a), but showed decreased survival from second line onwards, dropping to similar survival to adalimumab, with a lower adjusted drug survival than ustekinumab for third and subsequent lines of therapy; and lower than guselkumab and risankizumab for second and subsequent lines of therapy (Figure S2a–c). The adjusted drug survival at 2 years and the population‐averaged survival curves for the model stratified by lines of therapy are presented in Table S5 and Figure S2a–c, respectively.

### Sensitivity analyses

Sensitivity analyses revealed that prior treatment failure due to ineffectiveness with TNF‐a inhibitors, IL‐12/23 inhibitors or IL‐17 inhibitors was associated with a comparative decline in drug survival for people starting IL‐17 inhibitors (Figure S3a–c).

## DISCUSSION

We found that IL‐23p19 inhibitors (guselkumab and risankizumab) had the highest adjusted survival time for effectiveness, and both IL‐23p19 and IL12/23p40 inhibitors (ustekinumab) had the highest adjusted survival time for safety. We report for the first time the drug survival of brodalumab, finding it had similar drug survival for effectiveness compared to adalimumab and secukinumab, and for safety compared to the IL‐17A inhibitors and adalimumab. Prior biologic treatment and PsA led to varying effects for different biologics. Our study has importantly demonstrated that IL‐17R blockade results in similar persistence patterns to IL‐17A blockade, particularly in biologic‐experienced patients. This finding is clinically significant as it suggests that blocking the IL‐17R does not mitigate the loss of treatment response observed with prior biologic exposure. Additionally, this study presents the first full analysis comparing risankizumab and guselkumab in a large real‐world cohort (risankizumab *n* = 832 and guselkumab *n* = 1,258). While the observed differences in drug survival between these two IL‐23p19 inhibitors are minimal, this reinforces their similar efficacy and safety profiles, which is an important insight for clinicians selecting long‐term treatment options.

Consistent with our previous study, PsA and prior biologic exposures acted as effect modifiers.^5^ Ustekinumab showed decreased drug survival for PsA patients, likely due to comparatively lower treatment efficacy. All IL‐17 inhibitors showed a pronounced drop in drug survival with prior biologic failure compared to any other biologic class. We found that previous discontinuation due to ineffectiveness for TNF inhibitors, IL‐17 inhibitors and ustekinumab all resulted in a similar drop in drug survival for people starting on IL‐17 inhibitors. Contrary to our previous hypothesis, a broad inhibition of the IL‐17 cytokine family with IL‐17 receptor blockade, as is done by brodalumab, was not protective for a drop in drug survival with increasing lines of therapy; rather, brodalumab also showed a substantial drop in survival across increasing lines of therapy. Caution should be applied for the interpretation of these results, however, given the smaller sample size of people on brodalumab. Biologic‐experienced populations may reflect a selection of patients with more complex disease. Given that IL‐17 inhibitors target downstream effectors such as IL‐17A which influence keratinocytes directly without broad effects on upstream IL‐23 responsive cells such as Th17 and Tc17, patients with more complex disease may have psoriasis modulated by other effectors such as IL‐17F or IL‐22; a significant role for residual tissue resident memory cell‐17 or disease driven by upregulation of IL‐23 receptor, which are all targets of IL‐23 but not IL‐17A inhibition.^20^ Drug survival results for bimekizumab, an IL‐17A and F inhibitor, will help elucidate whether targeted additional inhibition of IL‐17F may help treatment effect for these patients.

This study's findings are consistent with the published literature.^6^, ^7^, ^8^, ^9^, ^10^, ^11^, ^12^, ^21^, ^22^, ^23^ A recent systematic review and meta‐analysis of 69 real‐world cohort studies aggregating drug survival outcomes of 48,704 psoriasis patients on IL‐23 and IL‐17 inhibitors revealed high drug survival rates, with the highest rates for guselkumab and risankizumab as seen in our study.^24^ Torres *et al.* conducted a multinational cohort study that included 549 and 693 treatment courses of brodalumab and risankizumab respectively and reported survival functions of 0.80 (95% CI 0.76–0.84) for brodalumab and 0.92 (95% CI 0.89–0.95) for risankizumab at 24 months, compared with 0.75 for secukinumab and 0.79 for ixekizumab at 24 months, with discontinuation restricted to those associated with ineffectiveness. In contrast to the present study, they found superior overall drug survival for brodalumab and ixekizumab when compared with secukinumab, which could be explained by the discrepancy between biologic‐naive patients on brodalumab between our study (19.9%) and Torres *et al.* (48.2%).^23^ Kojanova *et al*. analysed drug survival data of 949 patients on IL‐17 inhibitors from the Czech Republic BIOREP registry and found no significant differences in drug survival between brodalumab, ixekizumab and secukinumab, which is similar to our findings.^22^

One limitation in biologic drug survival studies is the restricted generalisability due to smaller sample sizes,^9^ single‐centre designs^6^, ^11^, ^21^ and limited follow‐up.^12^ This contrasts with our data from BADBIR, one of the largest prospective psoriasis registers, and in particular our study has a large sample of people on IL‐23p19 inhibitors, enabling us to make confident estimations of comparative drug survival. We also adjusted for multiple potential confounders to maximize robustness and reduce confounding bias, and we looked at subgroup effects, which are clinically important and contrast with other studies such as Torres *et al.* ^23^ We used the RMST as a clinically interpretable measure of the absolute difference in drug survival in the context of non‐proportional hazards between the biologics.

Consistent with other observational studies, there may be residual selection bias. Missing data is an important limitation, especially for the newer biologics, IL‐17A inhibitors and p19 inhibitors. There is a shorter follow‐up for newer biologics, which limits our ability to include RMST over longer periods. We were unable to consider dosing regimens; in particular, we had missing data for over 50% of dosing frequency data for secukinumab and had only 0.2% of entries classed as fortnightly administration, and therefore we were unable to evaluate the impact of this dosing regimen on treatment persistence. Our results are based on the UK and RoI healthcare system and may not be generalisable to other populations. The smaller brodalumab cohort and lack of tildrakizumab (IL‐23p19 inhibitor) data due to small sample size are additional limitations.

IL‐23 inhibitors may provide greater efficacy and durability compared to other biologic classes due to their targeted and modulating effect on Th17 regulatory immune cells leading to more complete suppression of inflammation with fewer off‐target effects.

Additionally, IL‐23 inhibitors sustain long‐term clinical responses even after treatment withdrawal, which is linked to continued suppression of the IL‐23 axis and its downstream effectors. This contrasts with IL‐17 inhibitors, which act downstream and upon drug withdrawal are associated with more rapid disease recurrence. Moreover, IL‐23 inhibition has been shown to favourably shift the balance of CD8^+^ tissue‐resident memory cells and T regulatory cells, which contributes to long‐lasting remission and reduced relapse rates.^25^

### Clinical implications

In individuals with psoriasis and no other significant comorbid conditions (e.g. axial spondyloarthritis), guselkumab and risankizumab offer good treatment longevity for both effect and safety and should be considered for people who have had previous biologic failures. In individuals without PsA, ustekinumab demonstrated superior drug survival compared to secukinumab and comparable drug survival to ixekizumab for effectiveness, and comparable safety to IL‐23p19 inhibitors. Given the imminent availability of ustekinumab as a biosimilar, it may potentially supersede adalimumab as the first‐line biologic treatment for cost‐effectiveness. For biologic‐naïve patients valuing treatment longevity, guselkumab, ixekizumab and risankizumab have similar persistence and are viable options.

## CONCLUSIONS

Drug survival is high with IL23p19 inhibitors. People with psoriasis persist with IL23p19 inhibitors up to an estimated 21 weeks more for effectiveness and 13 weeks more for safety compared with other biologics over a 2year period on average. Persistence with brodalumab is similar to adalimumab and secukinumab for effectiveness and similar to adalimumab, secukinumab and ixekizumab for safety. We identified PsA and previous biologic exposure as effect modifiers for treatment effectiveness, which were consistent findings from our previous studies. Having PsA has a detrimental effect on ustekinumab drug survival; therefore, it could impact clinician choice. Our study also suggests that treatment persistence declines over lines of therapy for secukinumab, ixekizumab, and brodalumab to a greater extent than ustekinumab, adalimumab, or the IL23p19 inhibitors. This evidence on the absolute difference in time persisted on biologic may help clinicians and patients make an informed decision on choosing the right biologic, including differentiating between different classes of biologics based on the patient's history of having PsA or not, their treatment history, and whether they prioritize treatment longevity.

## AUTHOR CONTRIBUTIONS

**Zenas Yiu:** Conceptualization, data curation, formal analysis, methodology, visualization, writing original draft (lead), review and editing (lead). **Leila Motedayen Aval:** Visualization, writing original draft (lead), review and editing (lead). **Oras Alabas, Christopher Griffiths, Nick Reynolds, Philip Hampton, Catherine H. Smith, Richard Warren:** Review and editing (supporting).

## FUNDING INFORMATION

British Association of Dermatologists Biologic Register Ltd. (BADBRL) is a registered company within the British Association of Dermatologists and is coordinated by The University of Manchester. BADBRL receives income from AbbVie, Almirall, Eli Lilly, J&J, Leo Pharma, Novartis, Samsung Bioepis, UCB, BI and BMS for providing pharmacovigilance services. The research was supported by the NIHR Manchester, Guy's and St Thomas' and Newcastle's Biomedical Research Centres. The views expressed are those of the authors and not necessarily those of the National Health Service, the NIHR or the Department of Health. All decisions concerning analysis, interpretation and publication are made independently of any industry contribution. All relevant information regarding serious adverse events mentioned in this publication has been reported to the appropriate company as per the contractual agreements/standard operating procedures. This research was supported by the NIHR Manchester Biomedical Research Centre (NIHR203308) and Department of Health via the NIHR BioResource Clinical Research Facility. C.E.M.G. is an Emeritus NIHR Senior Investigator. N.J.R. and PJH are supported by the NIHR Newcastle Biomedical Research Centre and the NIHR Newcastle Patient Safety Research Centre. N.J.R. is also supported by the NIHR Newcastle Health Tech Research Centre and is an NIHR Senior Investigator.

## CONFLICT OF INTEREST STATEMENT

N.J.R. reported research support from AbbVie, Boehringer Ingelheim and Celgene as well as grants from Novartis, UCB and PSORT (with multiple industry partners) outside the submitted work. C.H.S. reported grants from a Medical Research Council‐funded stratified medicine consortium with multiple industry partners, grants from an Innovative Medicines Initiative (Horizon 2020)‐funded European consortium with multiple industry partners and research grants from AbbVie, Novartis, Pfizer, Sanofi and Boehringer Ingelheim, outside the submitted work; she is also chair of UK guidelines on biologic therapy in psoriasis. R.B.W. reported personal fees from AbbVie, Almirall, Amgen, Arena, Astellas, Avillion, Boehringer Ingelheim, Biogen, Bristol Myers Squibb, Celgene, DiCE, GSK, Janssen, Leo Pharma, Lilly, Novartis, Sanofi, Sun Pharma, UCB and Union and grants from AbbVie, Almirall, Amgen, Celgene, Janssen, Novartis and UCB outside the submitted work. P.J.H. has received educational grants, consultancy fees and research funding from Janssen, AbbVie, Eli Lilly and LEO Pharma. C.E.M.G. reported grants from AbbVie, Amgen, Almirall, Boehringer Ingelheim, Anaptysbio and UCB Pharma as well as personal fees from AbbVie, Artax, Boehringer Ingelheim, Boots UK Ltd., Bristol Myers Squibb, Celltrion, Eli Lilly, GSK, Janssen, Kyowa Kirin, Novartis, ONO Pharmaceuticals and Sun Pharma during the conduct of the study. No other disclosures were reported.

## ETHICAL APPROVAL

BADBIR received approval by the National Health Service Research Ethics Committee North‐West England in March 2007 (07/MRE08/9).

## ETHICS STATEMENT

All patients provided written informed consent in accordance with the Declaration of Helsinki.

## Supporting information


Table S1.

Figure S1.

Figure S2.

Figure S3.

Appendix S1.

## Data Availability

The data underlying this article cannot be shared publicly owing to the linkages to other health resources.
