# Supplementary material for: Drug survival of IL‐23 and IL‐17 inhibitors versus other biologics for psoriasis: A British Association of Dermatologists Biologics and Immunomodulators Register cohort study
Source: J Eur Acad Dermatol Venereol. 2025 May 29;39(10):1785–95. doi: 10.1111/jdv.20739 (PMC12466084; doi:10.1111/jdv.20739)
Supplement: Supplementary file 1 — Table S1. [file JDV-39-1785-s001.docx]

**Supporting Information**

**Supplementary Table 1 Survival functions at years 1, 2, and 3 for the biologic cohorts stratified by reason for discontinuation,**

| **Reasons for drug discontinuation** | **Adalimumab** | | **Ustekinumab** | | **Secukinumab** | | **Ixekizumab** | | **Brodalumab** | | **Guselkumab** | | **Risankizumab** | |
| --- | --- | --- | --- | --- | --- | --- | --- | --- | --- | --- | --- | --- | --- | --- |
|  | **Total participants / no. discontinuations** | **Survival function (95% CI)** | **Total participants / no. discontinuations** | **Survival function (95% CI)** | **Total participants / no. discontinuations** | **Survival function (95% CI)** | **Total participants / no. discontinuations** | **Survival function (95% CI)** | **Total participants / no. discontinuations** | **Survival function (95% CI)** | **Total participants / no. discontinuations** | **Survival function (95% CI)** | **Total participants / no. discontinuations** | **Survival function (95% CI)** |
| **Overall** | | | | | | | | | | | | | | |
| **Year 1** | **4928/1695** | **0.75 (0.74,0.76)** | **4586/852** | **0.84 (0.84,0.85)** | **2283/540** | **0.81 (0.80,0.83)** | **686/174** | **0.82 (0.79,0.84)** | **240/101** | **0.71 (0.66,0.76)** | **860/142** | **0.87 (0.85,0.89)** | **400/64** | **0.90 (0.87,0.92)** |
| **Year 2** | **3728/979** | **0.59 (0.58,0.61)** | **3726/605** | **0.73 (0.72,0.74)** | **1672/424** | **0.66 (0.64,0.67)** | **410/130** | **0.64 (0.61,0.68)** | **154/32** | **0.60 (0.55,0.65)** | **566/63** | **0.80 (0.77,0.82)** | **154/23** | **0.82 (0.78,0.86)** |
| **Year 3** | **2893/628** | **0.49 (0.48,0.50)** | **3070/385** | **0.65 (0.64,0.67)** | **1157/307** | **0.53 (0.51,0.55)** | **243/70** | **0.52 (0.48,0.56)** | **90/22** | **0.50 (0.44,0.56)** | **302/36** | **0.73 (0.70,0.76)** | **28/9** | **0.73 (0.64,0.80)** |
| **Ineffectiveness** | | | | | | | | | | | | | | |
| **Year 1** | **4928/878** | **0.86 (0.85,0.87)** | **4586/461** | **0.91 (0.90,0.92)** | **2283/293** | **0.89 (0.88,0.90)** | **686/80** | **0.91 (0.89,0.93)** | **240/64** | **0.80 (0.76,0.84)** | **860/56** | **0.95 (0.93,0.96)** | **400/30** | **0.95 (0.93,0.97)** |
| **Year 2** | **3728/414** | **0.78 (0.77,0.79)** | **3726/295** | **0.85 (0.84,0.86)** | **1672/277** | **0.78 (0.76,0.79)** | **410/84** | **0.78 (0.75,0.81)** | **154/18** | **0.73 (0.68,0.78)** | **566/31** | **0.91 (0.89,0.92)** | **154/11** | **0.91 (0.88,0.94)** |
| **Year 3** | **2893/182** | **0.74 (0.73,0.75)** | **3070/178** | **0.81 (0.80,0.82)** | **1157/207** | **0.67 (0.65,0.69)** | **243/39** | **0.69 (0.65,0.73)** | **90/16** | **0.64 (0.57,0.70)** | **302/19** | **0.87 (0.84,0.89)** | **28/3** | **0.89 (0.85,0.92)** |
| **Adverse events** | | | | | | | | | | | | | | |
| **Year 1** | **4928/415** | **0.93 (0.93,0.94)** | **4586/255** | **0.96 (0.95,0.96)** | **2283/159** | **0.94 (0.93,0.95)** | **686/73** | **0.92 (0.90,0.94)** | **240/26** | **0.92 (0.88,0.94)** | **860/48** | **0.96 (0.94,0.97)** | **400/19** | **0.97 (0.95,0.98)** |
| **Year 2** | **3728/191** | **0.89 (0.88,0.90)** | **3726/151** | **0.92 (0.92,0.93)** | **1672/77** | **0.91 (0.89,0.92)** | **410/28** | **0.87 (0.85,0.90)** | **154/11** | **0.87 (0.82,0.90)** | **566/17** | **0.93 (0.92,0.95)** | **154/8** | **0.94 (0.91,0.96)** |
| **Year 3** | **2893/129** | **0.86 (0.85,0.87)** | **3070/89** | **0.90 (0.89,0.91)** | **1157/59** | **0.87 (0.86,0.88)** | **243/20** | **0.82 (0.79,0.85)** | **90/6** | **0.83 (0.77,0.87)** | **302/6** | **0.92 (0.90,0.94)** | **28/3** | **0.87 (0.77,0.93)** |

**Supplementary Table 2: Final multivariable flexible parametric survival model for drug discontinuation associated with ineffectiveness**

| **Covariate** | **Hazard ratio (95% CI)** |
| --- | --- |
| Age | 0.99 (0.99,0.99) |
| Female sex | 1.12 (1.06,1.19) |
| Baseline Psoriasis Area and Severity Index | 1.01 (1.01,1.01) |
| BMI | 0.05 (0.02,0.14) |
| Waist | 1.00 (1.00,1.01) |
| Palmoplantar psoriasis | 1.10 (1.02,1.19) |
| Diabetes | 1.20 (1.09,1.32) |
| COPD | 1.27 (1.04,1.55) |
| Flexural psoriasis | 1.06 (1.00,1.13) |
| Methotrexate use | 1.16 (1.05,1.30) |
| Cyclosporin use | 3.22 (2.77,3.74) |
| Psoriatic arthritis | 1.22 (1.08,1.37) |
| Nail involvement | 1.08 (1.01,1.15) |
| **Previous biologic experience** | |
| 1 previous biologic | 1.57 (1.39,1.77) |
| 2 previous biologics | 1.68 (1.44,1.95) |
| **Ethnicity** | |
| White | Ref |
| Asian | 1.20 (1.06,1.37) |
| Other | 0.96 (0.82,1.13) |
| **Biologic therapies** | |
| Ustekinumab | Ref |
| Adalimumab | 1.28 (1.13,1.45) |
| Secukinumab | 1.87 (1.56,2.26) |
| Ixekizumab | 0.88 (0.50,1.56) |
| Brodalumab | 0.75 (0.34,1.65) |
| Guselkumab | 0.43 (0.21,0.87) |
| Risankizumab | 0.41 (0.12,1.34) |
| Biologic therapies * number of previous biologics interaction terms | |
| Adalimumab * 1 previous biologic | 0.64 (0.54,0.75) |
| Secukinumab * 1 previous biologic | 0.99 (0.81,1.22) |
| Ixekizumab * 1 previous biologic | 1.38 (0.76,2.52) |
| Brodalumab * 1 previous biologic | 1.67 (0.74,3.80) |
| Guselkumab * 1 previous biologic | 1.03 (0.50,2.16) |
| Risankizumab * 1 previous biologic | 0.86 (0.32,2.27) |
| Adalimumab * 2 previous biologics | 0.94 (0.75,1.19) |
| Secukinumab * 2 previous biologics | 1.33 (1.06,1.67) |
| Ixekizumab * 2 previous biologics | 3.08 (1.77,5.38) |
| Brodalumab * 2 previous biologics | 2.40 (1.13,5.09) |
| Guselkumab * 2 previous biologics | 2.03 (1.05,3.93) |
| Risankizumab * 2 previous biologics | 1.27 (0.51,3.17) |
| **Biologic therapies * psoriatic arthritis interaction terms** | |
| Adalimumab | 0.78 (0.66,0.91) |
| Secukinumab | 0.84 (0.70,1.00) |
| Ixekizumab | 0.71 (0.53,0.96) |
| Brodalumab | 1.20 (0.79,1.84) |
| Guselkumab | 0.95 (0.63,1.44) |
| Risankizumab | 0.53 (0.23,1.21) |

Abbreviations: BMI, body mass index (calculated as weight in kilograms divided by height in meters squared); COPD, chronic obstructive pulmonary disease

a Comparisons of model fit statistics suggested that 5 knots and 2 knot be placed for the restricted cubic splines to model the baseline hazard and the time-dependent effect of biologic treatment, respectively. The effect estimates for restricted cubic splines are available on request.

b BMI transformation = (BMI/10)−2.

c Other ethnicity included Black British individuals.

**Supplementary Table 3: Final multivariable flexible parametric survival model for drug discontinuation associated with adverse events**

| **Covariate** | **Hazard ratio (95% CI)** |
| --- | --- |
| Age ((age/10^0.5^)) | 0.01 (0.00,0.04) |
| Age (age/10) | 3.45 (2.40,4.96) |
| Female sex | 1.53 (1.37,1.70) |
| Baseline Psoriasis Area and Severity Index | 1.00 (1.00,1.01) |
| BMI | 0.99 (0.98,1.00) |
| Waist | 1.01 (1.00,1.01) |
| Palmoplantar psoriasis | 1.20 (1.06,1.35) |
| Diabetes | 0.75 (0.63,0.90) |
| Unstable psoriasis | 1.19 (1.02,1.38) |
| Cyclosporin use | 1.53 (1.10,2.11) |
| Psoriatic arthritis | 0.32 (0.28,0.38) |
| Number of comorbid conditions | 1.16 (1.11,1.21) |
| Dyslipidaemia | 0.75 (0.63,0.90) |
| **Biologic therapies** | |
| Ustekinumab | Ref |
| Adalimumab | 1.16 (0.98,1.37) |
| Secukinumab | 1.28 (0.98,1.67) |
| Ixekizumab | 1.40 (0.82,2.40) |
| Brodalumab | 1.99 (0.79,5.04) |
| Guselkumab | 0.45 (0.22,0.90) |
| Risankizumab | 0.38 (0.02,6.99) |

Abbreviations: BMI, body mass index (calculated as weight in kilograms divided by height in meters squared); PsA, psoriatic arthritis.

a Comparisons of model fit statistics suggested that 5 knots and 2 knot be placed for the restricted cubic splines to model the baseline hazard and the time-dependent effect of biologic treatment, respectively. The effect estimates for restricted cubic splines are available on request.

**Supplementary Table 4 - Standardised difference (Cohen’s d) in restricted mean survival time at 2 years between biologics with and without psoriatic arthritis**

Psoriatic arthritis

| **Adalimumab** | **0.15 (0.07,0.22)** | **0.09 (0.02,0.17)** | **0.26 (0.16,0.37)** | **-0.12 (-0.32,0.09)** | **0.38 (0.31,0.45)** | **0.39 (0.22,0.55)** |
| --- | --- | --- | --- | --- | --- | --- |
| **-0.23 (-0.27,-0.19)** | **Ustekinumab** | **-0.06 (-0.14,0.02)** | **0.17 (0.06,0.28)** | **-0.33 (-0.53,-0.12)** | **0.29 (0.21,0.37)** | **0.35 (0.18,0.51)** |
| **-0.13 (-0.19, -0.08)** | **0.11 (0.06,0.17)** | **Secukinumab** | **0.22 (0.11,0.33)** | **-0.25 (-0.46,-0.04)** | **0.35 (0.27,0.43)** | **0.38 (0.21,0.55)** |
| **-0.29 (-0.31, -0.14)** | **-0.04 (-0.12,0.05)** | **-0.16 (-0.26,-0.06)** | **Ixekizumab** | **-0.49 (-0.71,-0.27)** | **0.13 (0.02,0.23)** | **0.21 (0.02,0.39)** |
| **-0.09 (-0.22,0.03)** | **0.16 (0.04,0.28)** | **0.04 (-0.08,0.17)** | **0.20 (0.05,0.34)** | **Brodalumab** | **0.69 (0.48,0.90)** | **0.60 (0.34,0.86)** |
| **-0.36 (-0.42,-0.30)** | **-0.22 (-0.28,-0.16)** | **-0.36 (-0.43,-0.28)** | **-0.22 (-0.31,-0.12)** | **-0.44 (-0.57,-0.30)** | **Guselkumab** | **0.09 (-0.08,0.26)** |
| **-0.32 (-0.40,-0.24)** | **-0.17 (0.26,-0.09)** | **-0.29 (-0.37,-0.20)** | **-0.14 (-0.25,-0.03)** | **-0.30 (-0.45,-0.16)** | **0.04 (-0.05,0.13)** | **Risankizumab** |

Each cell contains the standardised mean difference and 95% confidence interval between the restricted mean survival time over 2 years for the two outcomes of effectiveness and safety of the intervention in the respective column versus the comparator in the respective row. The cells shaded in green show superiority for the intervention; red shows inferiority for the intervention; and white shows no statistical difference between the intervention and the comparator.

**Supplementary Table 5 - Standardised difference (Cohen’s d) in restricted mean survival time at 2 years between biologics stratified by lines of therapy**

| **Adalimumab** |  |  |  |  |  |  |
| --- | --- | --- | --- | --- | --- | --- |
| **1st: -0.24 (-0.29,-0.19)**  **2nd:-0.12 (-0.19,-0.05)**  **≥3rd:-0.35 (-0.47,-0.24)** | **Ustekinumab** |  |  |  |  |  |
| **1st: -0.20 (-0.26,-0.13)**  **2nd: -0.05 (-0.13,0.03)**  **≥3rd: -0.10 (-0.21,0.02)** | **1st: -0.05 (-0.02,0.12)**  **2nd: 0.08 (0.00,0.15)**  **≥3rd: 0.24 (0.15,0.33)** | **Secukinumab** |  |  |  |  |
| **1st: -0.35 (-0.49,-0.21)**  **2nd:-0.23 (-0.35,-0.11)**  **≥3rd:-0.11 (-0.24,0.02)** | **1st: -0.19 (-0.34,-0.05)**  **2nd:-0.13 (-0.25,-0.02)**  **≥3rd: 0.21 (0.10,0.31)** | **1st: -0.26 (-0.41,-0.11)**  **2nd:-0.21 (-0.34,-0.09)**  **≥3rd:-0.02 (-0.13,0.09)** | **Ixekizumab** |  |  |  |
| **1st: -0.22  (-0.45,0.02)**  **2nd: 0.14 (-0.07,0.35)**  **≥3rd: 0.18 (0.01,0.34)** | **1st: -0.00  (-0.23,0.24)**  **2nd: 0.29 (0.08,0.50)**  **≥3rd: 0.57 (0.41,0.72)** | **1st: -0.05  (-0.28, 0.19)**  **2nd: 0.21 (-0.00,0.42)**  **≥3rd: 0.29 (0.14,0.44)** | **1st: 0.23 (-0.03,0.50)**  **2nd: 0.41 (0.18,0.64)**  **≥3rd: 0.27 (0.11,0.44)** | **Brodalumab** |  |  |
| **1st: -0.39 (-0.51,-0.27)**  **2nd:-0.39 (-0.50,-0.27)**  **≥3rd:-0.43 (-0.56,-0.31)** | **1st: -0.25 (-0.37,-0.13)**  **2nd:-0.30 (-0.41,-0.19)**  **≥3rd:-0.14 (-0.24,-0.03)** | **1st: -0.32 (-0.46,-0.19)**  **2nd:-0.39 (-0.51,-0.27)**  **≥3rd:-0.34 (-0.44,-0.24)** | **1st: -0.08 (-0.26,0.10)**  **2nd:-0.19 (-0.34,-0.05)**  **≥3rd:-0.30 (-0.42,-0.18)** | **1st: -0.31 (-0.57,-0.05)**  **2nd:-0.64 (-0.87,-0.41)**  **≥3rd:-0.61 (-0.77,-0.44)** | **Guselkumab** |  |
| **1st: -0.36 (-0.50,-0.22)**  **2nd:-0.38 (-0.50,-0.25)**  **≥3rd:-0.50 (-0.64,-0.35)** | **1st: -0.21 (-0.36,-0.07)**  **2nd:-0.30 (-0.42,-0.18)**  **≥3rd:-0.23 (-0.35,-0.10)** | **1st: -0.27 (-0.42,-0.12)**  **2nd:-0.38 (-0.51,-0.25)**  **≥3rd:-0.41 (-0.54,-0.29)** | **1st: -0.02 (-0.22,0.17)**  **2nd:-0.18 (-0.34,-0.02)**  **≥3rd:-0.36 (-0.50,-0.22)** | **1st: -0.21 (-0.48,0.06)**  **2nd:-0.57 (-0.81,-0.34)**  **≥3rd:-0.65 (-0.83,-0.47)** | **1st: 0.04 (-0.14,0.22)**  **2nd: 0.00 (-0.15,0.15)**  **≥3rd:-0.08 (-0.22,0.05)** | **Risankizumab** |

Each cell contains the standardised mean difference and 95% confidence interval between the restricted mean survival time over 2 years for the two outcomes of effectiveness and safety of the intervention in the respective column versus the comparator in the respective row. The numbers shaded in green show superiority for the intervention; red shows inferiority for the intervention; and black shows no statistical difference between the intervention and the comparator.
