# Supplementary material for: Drug survival of IL‐23 and IL‐17 inhibitors versus other biologics for psoriasis: A British Association of Dermatologists Biologics and Immunomodulators Register cohort study
Source: J Eur Acad Dermatol Venereol. 2025 May 29;39(10):1785–95. doi: 10.1111/jdv.20739 (PMC12466084; doi:10.1111/jdv.20739)
Supplement: Supplementary file 2 — Figure S1. [file JDV-39-1785-s002.pdf]

Supplementary Figure 1: Drug survival split by psoriatic arthritis (PsA) status.

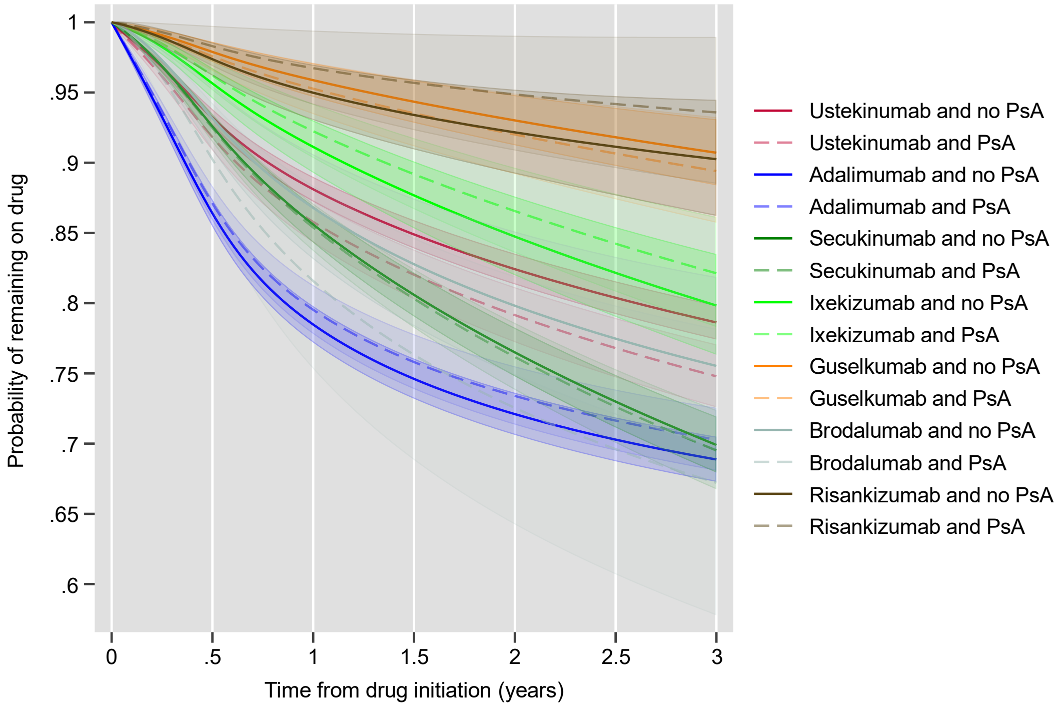

Population-averaged survival curves from the flexible parametric model (FPM) for all biologic therapies with or without psoriatic arthritis (PsA) for discontinuation. Shaded areas represent 95% confidence intervals from FPM curve. Note y-axis starts from 0.60 for presentation clarity purposes.
