# Supplementary material for: Drug survival of IL‐23 and IL‐17 inhibitors versus other biologics for psoriasis: A British Association of Dermatologists Biologics and Immunomodulators Register cohort study
Source: J Eur Acad Dermatol Venereol. 2025 May 29;39(10):1785–95. doi: 10.1111/jdv.20739 (PMC12466084; doi:10.1111/jdv.20739)
Supplement: Supplementary file 3 — Figure S2. [file JDV-39-1785-s003.pdf]

Supplementary Figure 2: **Overlaid Kaplan-Meier (KM) drug survival curves and the population-averaged Flexible Parametric Model (FPM) survival curves for the biologic therapies of a) first-line, b) second-line, and c) third or subsequent lines for discontinuation during 2 years.**

2a)

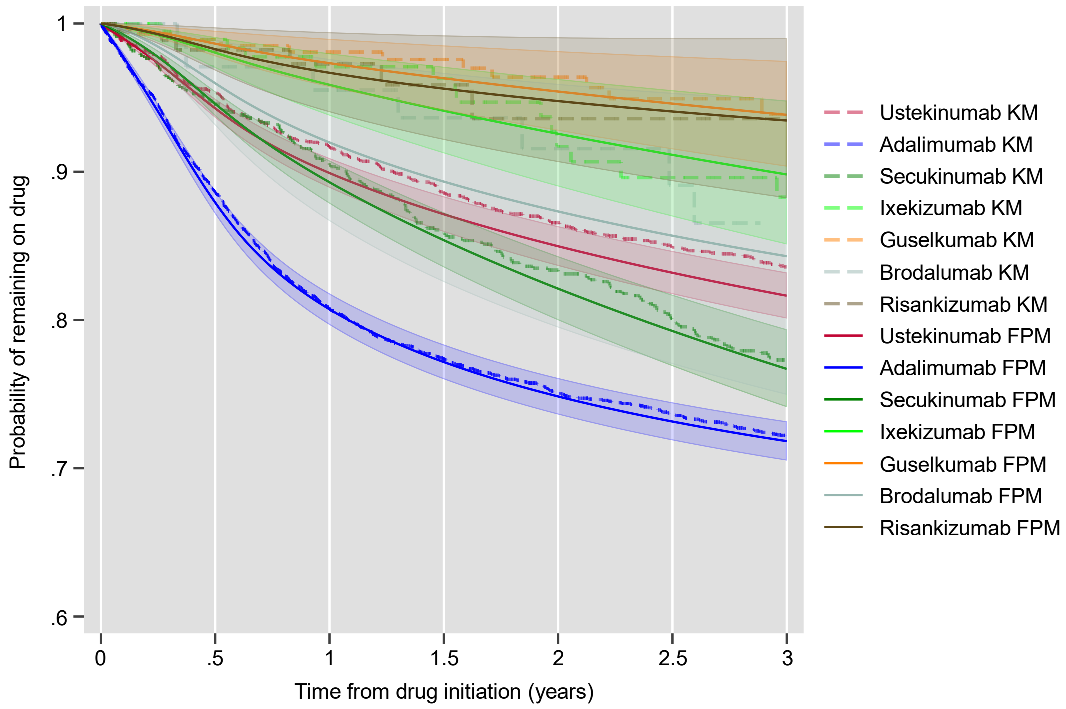

2b)

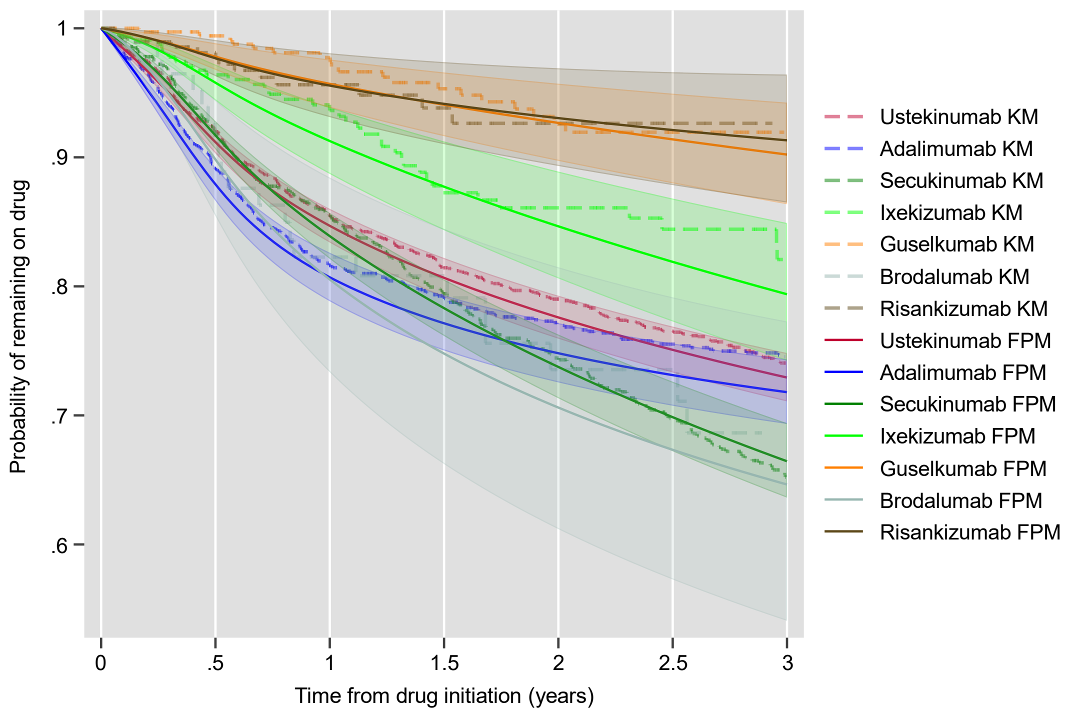

1
.9
.8
.7
.6
Probability of remaining on drug
0
.5
1
1.5
2
2.5
3
Time from drug initiation (years)
Ustekinumab KM
Adalimumab KM
Secukinumab KM
Ixekizumab KM
Guselkumab KM
Brodalumab KM
Risankizumab KM
Ustekinumab FPM
Adalimumab FPM
Secukinumab FPM
Ixekizumab FPM
Guselkumab FPM
Brodalumab FPM
Risankizumab FPM

2c)

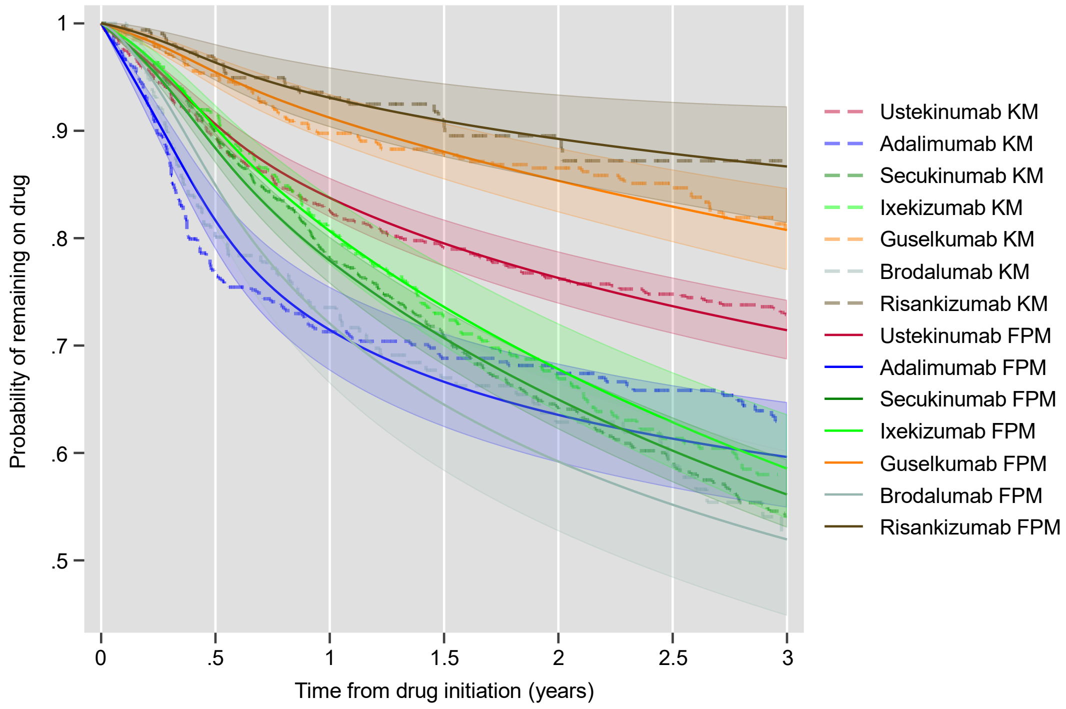

Note Y-axis starts from 0.6 in 2a) and 2b) and from 0.5 in 2c) for clarity purposes.
