# Supplementary material for: Drug survival of IL‐23 and IL‐17 inhibitors versus other biologics for psoriasis: A British Association of Dermatologists Biologics and Immunomodulators Register cohort study
Source: J Eur Acad Dermatol Venereol. 2025 May 29;39(10):1785–95. doi: 10.1111/jdv.20739 (PMC12466084; doi:10.1111/jdv.20739)
Supplement: Supplementary file 4 — Figure S3. [file JDV-39-1785-s005.pdf]

Supplementary Figure 3: **Sensitivity analysis looking at third or subsequent lines of therapy, and whether there is any difference by previous TNF-a inhibitor (a), ustekinumab (b), or IL-17 inhibitor (c) failure.**

3a)

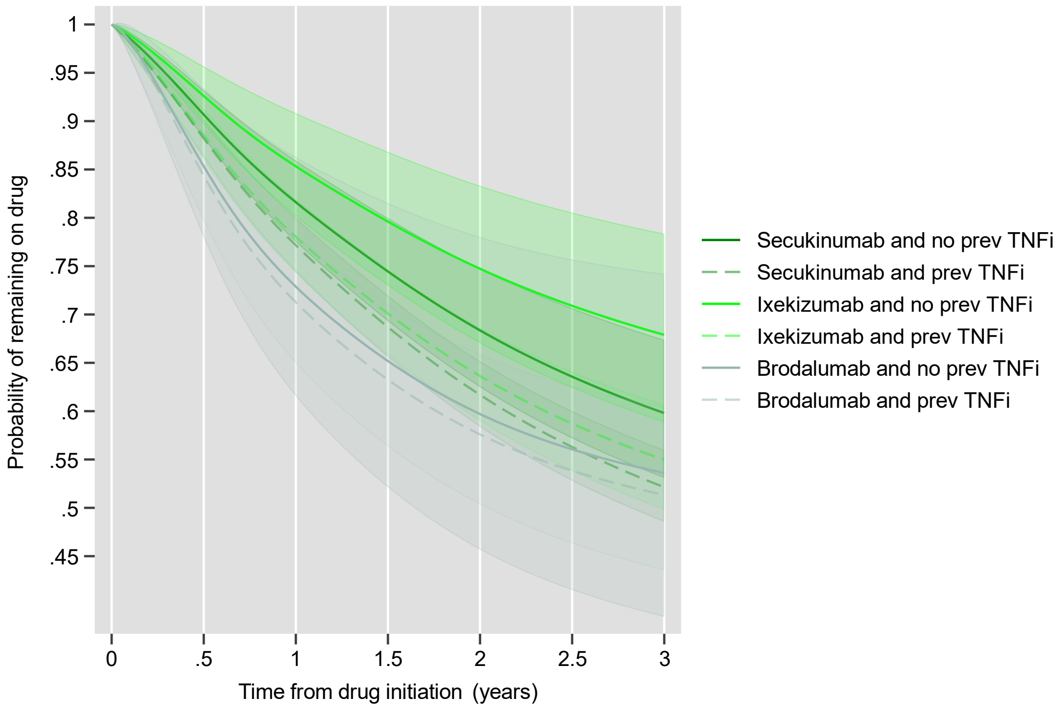

3b)

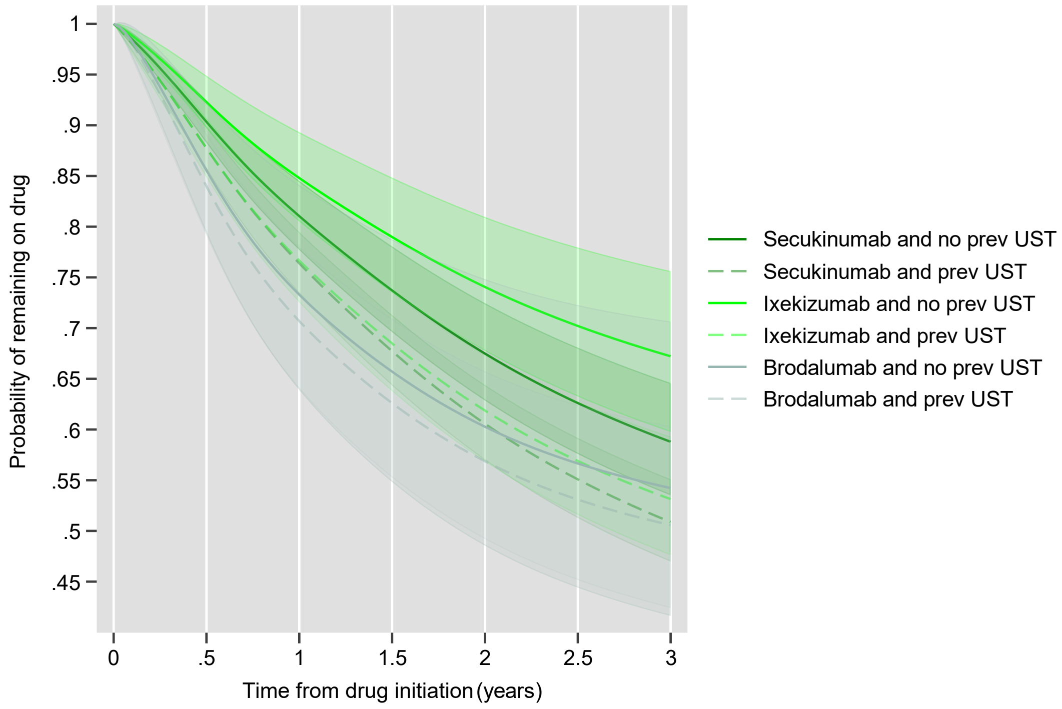

Probability of remaining on drug
1
.95
.9
.85
.8
.75
.7
.65
.6
.55
.5
.45
0
.5
1
1.5
2
2.5
3
Time from drug initiation (years)
Secukinumab and no prev UST
Secukinumab and prev UST
Ixekizumab and no prev UST
Ixekizumab and prev UST
Brodalumab and no prev UST
Brodalumab and prev UST

3c)

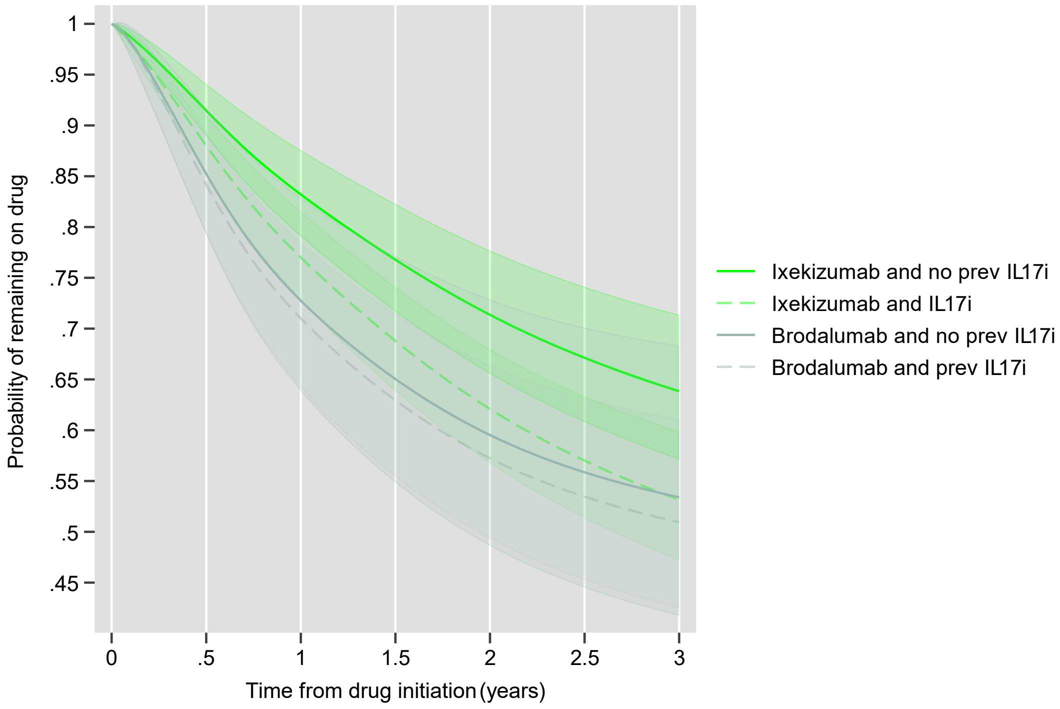

Note Y-axis starts from 0.45 for clarity purposes.
