# Supplementary material for: Drug survival of IL‐23 and IL‐17 inhibitors versus other biologics for psoriasis: A British Association of Dermatologists Biologics and Immunomodulators Register cohort study
Source: J Eur Acad Dermatol Venereol. 2025 May 29;39(10):1785–95. doi: 10.1111/jdv.20739 (PMC12466084; doi:10.1111/jdv.20739)
Supplement: Supplementary file 5 — Appendix S1. [file JDV-39-1785-s004.docx]

**Supporting Information**

**Collaborators:** BADBIR Study Group: Members of the British Association of Dermatologists Biologics and Immunomodulators Register (BADBIR) Study Group includes Philip Laws (Chair), Richard Warren (Chief Investigator), Shehnaz Ahmed, Oras Alabas, Zin Mon, Jonathan Barker, Anthony Bewley, Danielle Bowerbank, Chris Griffiths, Phil Hampton, Olivia Hughes, Brian Kirby, Elise Kleyn, Teena Mackenzie, Kathy McElhone, Tess McPherson, Simon Morrison, Nick Reynolds, Alexa Shipman, Catherine Smith, Shernaz Walton, Christina Ye, and Zenas Yiu. BADBIR Data Monitoring Committee consists of Anja Strangfeld (chair); Richard Weller; Girish Gupta and Vera Zietemann.
